# An In Vitro Approach to Model EMT in Breast Cancer

**DOI:** 10.3390/ijms24097757

**Published:** 2023-04-24

**Authors:** Lorenz Isert, Aditi Mehta, Gabriele Loiudice, Altea Oliva, Andreas Roidl, Olivia M. Merkel

**Affiliations:** 1 Pharmaceutical Technology and Biopharmacy, Ludwig-Maximilians-Universität München, Butenandtstr. 5-13, 81377 Munich, Germany; 2 Pharmaceutical Biotechnology, Ludwig-Maximilians-Universität München, Butenandtstr. 5-13, 81377 Munich, Germany

**Keywords:** breast cancer, EMT, tumor microenvironment, collagen coatings, EMT-phenotyping, shape factors

## Abstract

During the progression from ductal carcinoma in situ (DCIS) to invasive breast cancer (IBC), cells must overcome the physically restraining basement membrane (BM), which compartmentalizes the epithelium from the stroma. Since the extracellular matrix (ECM) of the epithelial and stromal compartments are biochemically and physically distinct from one another, the progression demands a certain degree of cellular plasticity for a primary tumor to become invasive. The epithelial-to-mesenchymal transition (EMT) depicts such a cell program, equipping cancer cells with features allowing for dissemination from the epithelial entity and stromal invasion at the single-cell level. Here, the reciprocal interference between an altering tumor microenvironment and the EMT phenotype was investigated in vitro. BM-typical collagen IV and stroma-typical collagen I coatings were applied as provisional 2D matrices. Pro-inflammatory growth factors were introduced to improve tissue mimicry. Whereas the growth on coated surfaces only slightly affected the EMT phenotype, the combinatorial action of collagen with growth factor TGF-β1 induced prominent phenotypic changes. However, EMT induction was independent of collagen type, and cellular accessibility for EMT-like changes was strongly cell-line dependent. Summarizing the entire body of data, an EMT-phenotyping model was used to determine cellular EMT status and estimate EMT-like changes. The miR200c-mediated reversion of mesenchymal MDA-MB-231 cells is reflected by our EMT-phenotype model, thus emphasizing its potential to predict the therapeutic efficacy of EMT-targeting drugs in the future.

## 1. Introduction

The epithelial-to-mesenchymal transition has been known to be a crucial part of embryogenesis for nearly half a century, but its critical role in cancer metastasis was revealed only recently in the early 2000s [1,2]. Since then, continuous and increasing interest in understanding the role of EMT in cancer metastasis has been reflected in about 6000 publications in 2019 [3]. While the role of EMT and its relevance during and for metastasis is still being discussed [4], three key features are commonly attributed to EMT or EMT-like changes.

First, the loss of proteins characterizing an epithelial phenotype and the acquisition of mesenchymal proteins is considered the basis of EMT (EMT hallmarks) [5]. Second, driven by signals received from the tumor microenvironment (TME) [6], EMT-relevant transcription factors (e.g., SNAIL, SLUG, TWIST) downregulate epithelial and/or upregulate mesenchymal genes that cause the re-organization of the cell cytoskeleton [1]. The resultant phenotype changes from a cobblestone-like epithelial morphology as the cell–cell junctions are abrogated and cells adopt a more spindle-like, elongated shape with a front–back polarity (Morphology). Third, as a consequence of epithelial depletions and mesenchymal fortifications, cellular motility is highly increased (Motility). An additional feature is the enhanced secretion of extracellular matrix (ECM)-degrading enzymes, promoting motility and helping cells to better cope with the migration and invasion that accompanies metastasis [7].

Importantly, EMT can be understood as a concept of cellular plasticity (epithelial-to-mesenchymal plasticity (EMP)) and adaptability. It is a reversible and non-binary transition that is not necessarily completed but rather partially fulfilled (partial EMT) [3,8]. Intermediate hybrid states (E/M states) possess both epithelial and mesenchymal features, and the degree of transition is governed by the contextuality of signaling within the tumor microenvironment, the developmental lineage of the distinct cancer types and (epi-) genetic alterations and regulations [3,9,10,11]. This trans-differentiation between epithelial and mesenchymal phenotypes has been described for many different kinds of carcinomas [12]. Aside from lung cancer, colorectal cancer, hepatocellular carcinoma and prostate cancer, breast cancer (BC) has been tremendously studied and proven clinically relevant in the context of EMT [12,13,14,15,16]. Based on gene expression profiling and clinical outcomes, breast cancer can be classified into four intrinsic subtypes with specific molecular marker expressions and increasing malignancies [17]. Luminal A and Luminal B subtypes express the estrogen receptor (ER) and the progesterone receptor (PR) (Luminal A > Luminal B), and both subtypes are fairly sensitive to endocrine therapies. The HER2 subtype lacks the latter sensitivity (being ER/PR negative) but displays an overexpression of human epidermal growth factor receptor 2 (HER2) [18,19]. The absence of ER and HER2 is descriptive of the basal-like subtype, which is considered a phenotype with a high mutation load and poor prognosis [20,21]. Triple-negative breast cancer (TNBC), a subgroup representing 70–80% of basal-like breast cancers, is characterized as negative for ER, PR and HER2 [19,22]. It is the most aggressive form of breast cancer due to a synergism of poor treatment options and a high metastatic potential, which presumably relies on EMT-like changes enabling DCIS-to-IBC progression [23,24,25].

The transmembrane protein E-cadherin can be considered “the guardian” of an epithelial phenotype. The extracellular domains of E-cadherin of each cell entangle with the extracellular domains of neighboring cells, leading to the establishment of adherens junctions [26]. Once downregulated, it is not only the physical/mechanical rupture that dissolves the epithelial phenotype. Intracellularly bound β-catenin (within the cytoplasmic cell adhesion complex) can translocate into the nucleus once E-cadherin is internalized and act as a transcription factor towards EMT [27,28].

It is well accepted that a switch in cellular intermediate filament (IF) usage from cytokeratin to vimentin occurs during EMT [3,29]. Vimentin is a network-forming type III intermediate filament and may be considered as the counterpart to E-cadherin, also because its expression is mainly restricted to mesenchymal cells [30,31]. By maintaining cytoskeletal integrity and mechanical strength, vimentin cushions traction stress during single-cell migration [32]. Apart from this load-bearing function, vimentin promotes microtubule polarity, which is a prerequisite for directed cell migration [30,33]. Vimentin IF (VIF) maturation depends on microtubular transport. Whilst providing the infrastructure for VIF network assembly, a long-lasting template of the microtubule’s architecture is simultaneously formed. Considering the fast turnover of microtubules (10 times faster than VIF), it appears that vimentin’s “memory” function eventually guides and enables persistent microtubule-mediated cell polarization and, consequently, directional migration [34].

For in vitro studies, the EMT machinery can be induced by extracellular stimuli in multiple ways, mainly by soluble factors like TGF-β, EGF or HGF [5,35]. However, it has been shown that solid components of the ECM trigger EMT in lung cancer. Cells, cultured on a type I collagen gel, activated autocrine TGF-β3 signaling, which in turn induced EMT, which is based on collagen I fiber recognition via integrins [35]. Likewise, Carey et al. [36] created a 3D collagen I matrix with defined mechanical properties, mimicking the stroma of the mammary gland. The incorporation of non-malignant breast epithelial cells into this scaffold upregulated mesenchymal genes, which was attributed to both biochemical and mechanical stimuli of the matrix. Interestingly, insertion of the same cell line into a basement-membrane-mimicking Matrigel (containing mainly collagen IV and laminin) did not provoke EMT-like changes. The authors concluded that the distinct ECM composition of epithelial (Matrigel) and stromal tissue (collagen I) differed between EMT occurring or not [36]. Furthermore, using a xenograft breast cancer mouse model, Vidal et al. reported that only cancer cells at the interface of the tumor and its stroma, i.e., cells that are directly exposed to the ECM of the stromal compartment, express vimentin, whereas cells in the core region of the tumor maintain cytokeratin expression [37]. Taken together, it appears that the signals cancer cells receive from the distinct ECM comprised within the epithelial (DCIS) and stromal (IBC) compartment ultimately dictate the present phenotype and phenotypic changes. It remains unknown to what extent the combinatorial action of solid and soluble factors in each compartment participate during initial as well as sustained EMT induction.

**Figure 1:**
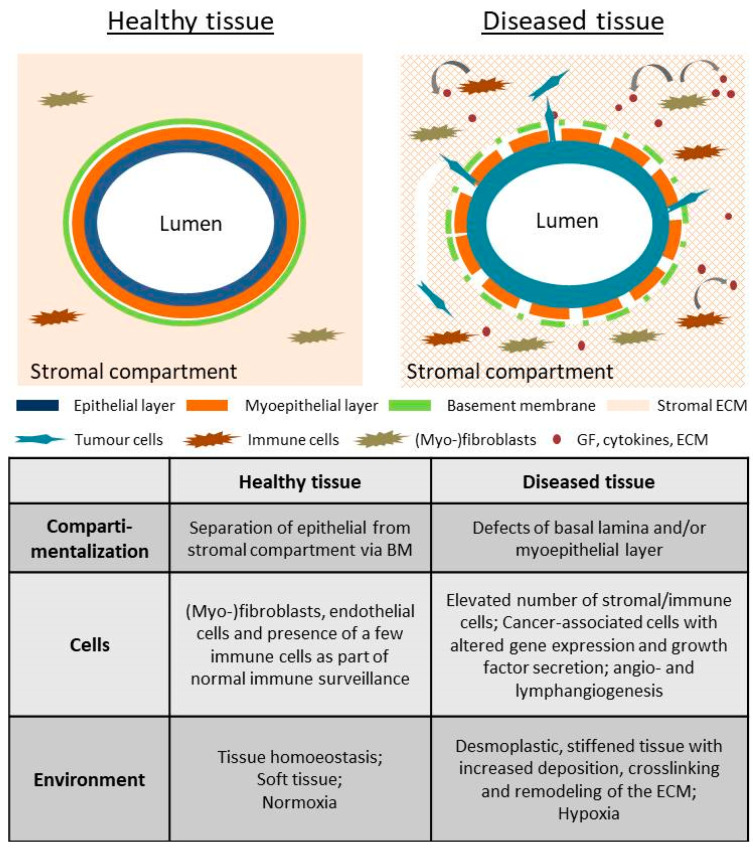
Simplified schematic cross-section of breast tissue including a mammary duct. The left panel shows the healthy tissue of the mammary gland. The mammary duct is formed by an epithelial layer (blue line) surrounded by myoepithelial cells (orange line) that are framed by the basement membrane (green line). The stromal compartment is well separated from the epithelium, which is also still the case for DCIS. The right panel demonstrates tissue alteration as part of tumor progression into IBC. Defects in the basement membrane (green dotted line) and/or myoepithelial layer (orange dotted line) occur. Stromal and epithelial compartments are exposed to each other, and cancer cells start to invade the stroma. The table below summarizes the most prominent differences between healthy and diseased tissue.

In this study, two common collagen coatings (globular type IV collagen and fibrillar type I collagen) were used as provisional matrices to depict the two opposing ECM components in the mammary gland (Figure 1). Globular type IV collagen is used as a part of the basement membrane (BM), the physiological substrate of the (myo-) epithelial layer. As described above, type I collagen is the main extracellular compound within the stromal fraction. In healthy tissue, it is well separated from the epithelium via the BM (Figure 1), but leakage during tumor progression causes cell exposure to it.

To stress and assess the concept of “contextuality of signaling” within the TME and its importance for EMT [4,10], breast cancer cells of distinct intrinsic subtypes were subjected to combinatorial treatment with different collagens and the prominent EMT-inducers TGF-β1 or EGF. Single treatment with either soluble or solid ECM components served as a reference. Initially, we defined the EMT status of the four breast cancer cell lines based on morphological aspects, EMT-marker (E-cadherin, CDH1 and Vimentin, VIM) expression and migratory behavior. MCF7 (Luminal A) and HCC1954 (HER2) cells were found to exhibit strong epithelial characteristics, whereas the MDA-MB-231 cell line (TNBC) was clearly attributed to a mesenchymal phenotype. The second TNBC cell line, MDA-MB-468, featured both epithelial and mesenchymal characteristics (epithelial > mesenchymal) and therefore was considered an E/M-hybrid. We then monitored the three mentioned EMT features under exposure to a simplified model, taking MDA-MB-231 attributes as a positive control/reference. Cellular shape factor image analysis served as a powerful tool to predict EMT-like changes. Together with the input from protein and migration analysis, a computational EMT model was created to further understand the complexity of EMT. Besides using the model to predict the EMT phenotype and phenotypic changes that arise during cellular stimulations, we exemplified its possible relevance in a miR200c-inducible cell line for the facile evaluation of therapeutic efficacy of new drug candidates targeting EMT.

## 2. Results

### 2.1. EMT Status

#### 2.1.1. EMT Marker

**Figure 2:**
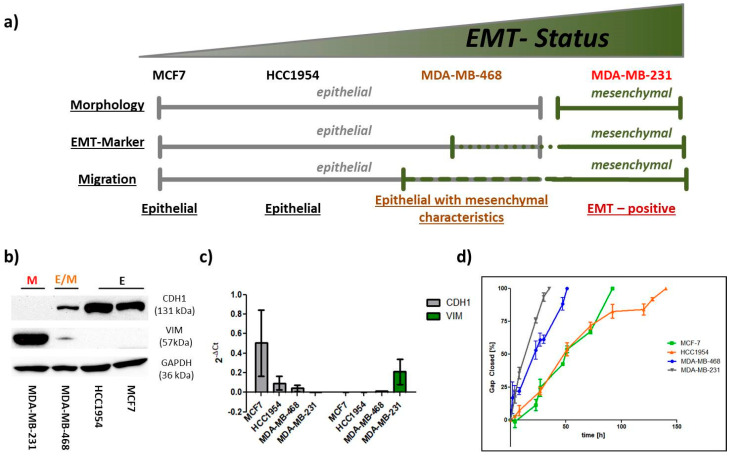
Defining the EMT status of the 4 breast cancer cell lines. (**a**) Quantifying cellular morphology, EMT-marker expression and cellular motility was used to define the cellular EMT status of each cell line. The scheme summarizes the outcome of the investigation, where grey lines indicate epithelial and green lines represent mesenchymal characteristics. Accordingly, MCF7 and HC1954 cell lines were shown to have epithelial (E) characteristics throughout the experiments. MDA-MB-468 cells were considered an E/M-hybrid cell line. The MDA-MB-231 cell line consistently demonstrated mesenchymal (M) characteristics and is considered an EMT-positive cell line. (**b**) Protein levels of CDH1, VIM and GAPDH (housekeeping) of the 4 cell lines. (**c**) mRNA expression of CDH1 and VIM gene are shown as 2^−∆Ct^, normalized to GAPDH mRNA expression. (**d**) Ibidi^®^ migration assay performed on 4 BC cell lines. Migration was analyzed by comparing the percentage of gap closure [%] over time (h). Area/time point was calculated based on three marked spots within each gap (n = 2). Error bars represent standard deviation (SD).

EMT is characterized by phenotypic changes in protein expression relying on extrinsic (GFs, hypoxia, ECM) and intrinsic (epigenetic) regulation [38,39]. To define the EMT status of our four breast cancer cell lines (Figure 2a), assessing the expression of phenotypic protein markers (EMT markers) is of fundamental importance. As described above, high levels of CDH1 are representative of an epithelial phenotype, whereas expression of VIM is a mesenchymal attribute. To this end, we performed Western blotting and qPCR analysis of the four breast cancer cell lines MCF7, HCC1954, MDA-MB-468 and MDA-MB-231 (Figure 2b,c). Both the Luminal A and HER2-positive cell lines strongly expressed E-cadherin, whereas they lacked vimentin expression. A considerably lower level of E-cadherin was detected for MDA-MB-468 (two-fold lower than for MCF7), but they co-expressed VIM. Furthermore, MDA-MB-231 was the only cell line to exhibit high levels of VIM, showing an absence of CDH1. We confirmed that the intrinsic subtype nomenclature for breast cancer matches with EMT-marker expression, and malignancy correlates with less epithelial but rather mesenchymal phenotypic marker expression. To improve this still binary system, we decided to take additional aspects into account to define the EMT status.

#### 2.1.2. Migration

Cellular migration is strongly dependent on cell–cell and cell–ECM adhesion [40]. Considering transcriptional repression of CDH1 and other cell–cell adhesion molecules as part of EMT, it appears logical that the resultant phenotype exhibits increased motility. VIM and cytoskeletal-dependent cell polarization further enhance the migratory ability of cancer cells during the transition. Thus, to estimate the cellular EMT status, we also evaluated the migratory behavior of the different cell lines using a wound-healing assay. Migration potential was quantified by means of percentage gap-closure over time, starting from 0% at t0. Figure 2d shows that MCF7 and HCC1954 cells were able to close the gap at a similar rate. On the other hand, the other two cell lines, MDA-MB-468 and MDA-MB-231, were two and three times faster, respectively. In a spheroid-based migration assay, we confirmed that MDA-MB-231 cells, which fail to express CDH1, spread significantly faster (5–10 times) than the CDH1-positive cell line MCF7 (Supplementary data, Figure S1).

#### 2.1.3. Morphology

Another feature of EMT is the re-structuring of the cytoskeleton based on spatiotemporal organization of actomyosin, microtubules, IF and other functional proteins [27,33,41,42]. In this process, the intrinsic mechanical properties of cells are altered, which leads to changes in cellular shape [43,44]. Actin filament polymerization is a driving force for the switch from a basolateral to a front–back polarity during EMT [45,46]. Fluorescently labeling the actin cytoskeleton allows for monitoring changes in cellular shape via confocal microscopy. To describe morphological changes in cancer cells, the aspect ratio A_R_, the cellular minor axis divided by its major axis (A_R_ = dmin/dmax), has been used [47,48,49,50]. In the context of EMT, a value close to 1 is attributed to an epithelial, cobblestone-like morphology, whereas values close to 0 describe a spindle-like appearance. We further evaluated nuclear pleomorphisms via nuclear circularity (C_N_). Nuclear pleomorphisms have been established to be clinically relevant in diseased tissues [51,52], but unfortunately, standard 2D in vitro cell culture results are not often able to link circularity to phenotypic changes [51,53]. Since nuclear envelop proteins are directly physically entangled with cytoskeletal proteins and nuclear dynamics are connected to cytoskeleton-mediated migration [54,55], we expected changes in nuclear circularity during EMT progression.

**Figure 3:**
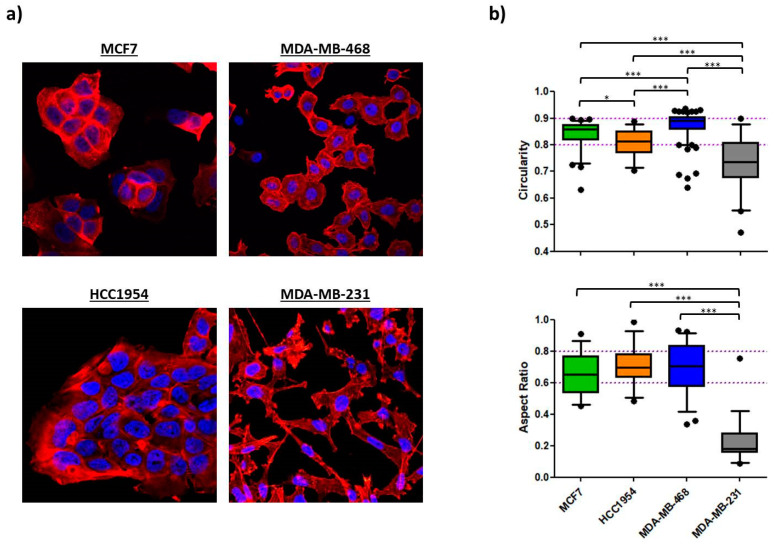
Morphological analysis of the 4 BC cell lines. (**a**) Confocal images of fixed cells recorded with a 63× objective. Nuclei are shown in blue (DAPI) and the actin cytoskeleton in red (TRITC). Cellular clustering and cuboidal shapes are typical epithelial features as shown for MCF7, HCC1954 and MDA-MB-468. F-actin was predominantly organized in cortical bundles tightly associated with cell–cell adhesions. Mesenchymal MDA-MB-231 cells exhibited spindle-shaped morphologies and failed to form cellular islets. (**b**) Image analysis via Fiji software is presented as whisker plots with 5–95 percentiles. The upper panel shows nuclear circularity C_N_ of the 4 cell lines (*n_nuclei_* = 33–74). Nuclear circularities between 0.8 and 0.9 were assigned to epithelial cells (dotted lines). The lower panel shows the cellular aspect ratio A_R_ of the 4 cell lines (*n_cells_* = 25). A_R_ values from 0.6 to 0.8 were attributed to epithelial cell shapes as indicated by the dotted lines. One-way ANOVA with a Bonferroni multiple comparison test was performed in GraphPad Prism software (Graph Pad Software, La Jolla, CA, USA) to calculate *p*-values (* *p* < 0.05, *** *p* < 0.001).

Confocal imaging revealed pronounced clustering of cells for MCF7, HCC1954 and MDA-MB-468 cells, even though the latter cells appeared to bundle less tightly (Figure 3). MDA-MB-231 did not cluster at all, showing an elongated shape. The aspect ratio of MDA-MB-231 (0.240 ± 0.157) was significantly lower in comparison to the other three cell lines, MCF7 (0.654 ± 0.136), HCC1954 (0.707 ± 0.128) and MDA-MB-468 (0.683 ± 0.170), which showed no significant differences amongst each other. A similar tendency was observed for the morphological assessment of the nucleus. Here, a circularity of 1 matches a perfect circle, and decreasing values describe progression to ellipsoid shapes. The nuclei of MDA-MB-231 had the most ellipsoid shapes (0.735 ± 0.101), which significantly differed from the other cell lines. Trending mean values of 0.808 ± 0.053 for HCC1954 and 0.877 ± 0.046 for MDA-MB-468 implied a stronger heterogeneity of nuclear circularity amongst the other three cell lines compared to the A_R_ value.

### 2.2. EMT Induction

To understand which factors potentially play a role in EMT induction in the mammary gland, it is crucial to become acquainted with the tissue’s architecture. As shown in Figure 1, the epithelium of the mammary duct comprises a layer of milk-producing luminal cells surrounded by myoepithelial cells. Apart from their contractile competence during lactation, myoepithelial cells produce the substrate of the epithelium, the basement membrane. This thin but dense layer consists of collagen IV, laminin and proteoglycans and is the border between the epithelial and stromal compartments [6]. The ducts are encircled by the microenvironment, comprising ECM, predominantly collagen I, and stromal cells (e.g., fibroblasts, endothelial cells, leukocytes). In healthy tissues and DCIS, the luminal epithelial cells are not in contact with the environment of the stromal compartment. It is not until the breakage of the BM by an invading tumor that the epithelial cancer cells come in contact with collagen I and the multitude of factors (growth factors, cytokines and enzymes) secreted by cancer-associated cells during tumor progression [56,57,58,59]. It is strikingly evident that the resultant inflamed and desmoplastic stroma (Figure 1) bears a high potential to induce EMT.

#### 2.2.1. EMT Marker

**Figure 4:**
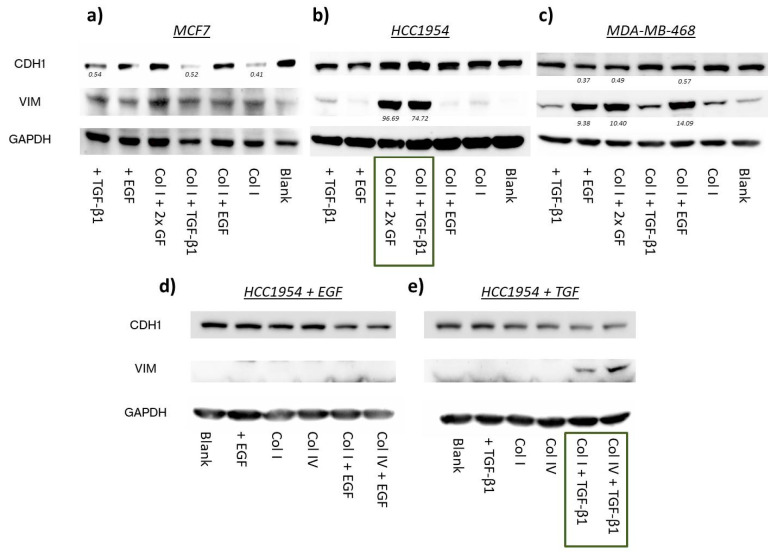
Protein levels of CDH1, VIM and GAPDH (housekeeping) were assessed via Western blotting from 30 µg of the total protein extracts of MCF7, HCC1954 and MDA-MB-468 cell line as part of the EMT-induction study. Cells were subjected to multiple treatments as indicated. Numbers in the blots indicate fold-changes in protein levels normalized to untreated control cells (Blank) after 72 h incubation. EMT induction study in MCF7 (**a**); HCC1954 (**b**); and MDA-MB-468 (**c**,**d**) EGF stimulation in HCC1954 dependent on collagen type; (**e**) TGF-β1 stimulation in HCC1954 dependent on collagen type.

To estimate the impact of the acellular stromal fraction of the mammary gland on our EMT model, we confronted the three cell lines with collagen I (as a major ECM component of the stroma) and the growth factors EGF and TGF-β1, respectively. In addition, attempts to further provoke EMT-like changes by combinatorial stimulation with all factors (Figure 4) were included.

MCF7 only showed minor changes in protein marker expression in comparison to untreated cells after 72 h incubation (Figure 4a). Normalization with the housekeeping protein revealed that CDH1 protein levels of cells grown on collagen I were reduced to a similar extent as in the TGF-β1 treatment (0.41 vs. 0.54). EGF appeared to have no impact on the EMT markers; rather, it abolished the effect of collagen I and TGF-β1 on CDH1 as part of a co-treatment. Combinatorial stimulation with collagen I and TGF-β1 did not further decrease E-cadherin expression (0.52). On the contrary, none of the treatments affected VIM levels.

Protein levels of the HCC1954 cell line demonstrated the importance of the concept of contextuality of signaling for EMT induction (Figure 4b). Single treatments and treatment of collagen I with EGF were inefficient in mediating phenotypic marker changes. Interestingly, the co-action of TGF-β1 with collagen I did substantially enhance VIM protein expression (75-fold). This was also true when EGF was additionally added to the latter two. However, CDH1 protein levels were not strongly altered. Taken together, it appears that the growth on collagen I allowed for TGF-β1 to unfold its EMT-inducing capacities even if it only caused an incomplete pEMT.

In accordance with what has been reported elsewhere [60], EGF stimulation was able to drive EMT in MDA-MB-468 cells, as demonstrated in Figure 4c. Both CDH1 and VIM protein levels underwent EMT-typical changes. VIM was highly upregulated (9.4-fold) and CDH1 levels faded considerably (0.37). Combinatorial treatments with EGF vaguely fostered EMT induction in MDA-MB-468. A minor increase in VIM protein levels was observed under treatment with collagen I or TGF-β1 or with both.

As described above, invading tumors must face the influence of collagen I once they have degraded the BM and enter the stromal compartment. Growing in situ carcinomas arising from luminal epithelial cells eventually interfere with collagen IV within the BM as they displace myoepithelial cells or the latter are depleted as a result of tumor progression [56,57]. Consequently, examining the impact that collagen IV exerted on cancer cells under exposure to growth factors and comparing the outcome to growth on collagen I is of interest here.

Therefore, we included collagen IV in our EMT-marker study. Trilateral treatments (TGF-β1 + EGF + collagen) were excluded, as they seemed to have no additive effect on EMT induction. For MCF7 and MDA-MB-468, we confirmed the phenotypic changes of EMT-protein levels of earlier findings (Supplementary data, Figure S2). In MCF7, after normalization, E-cadherin levels decreased for cell growth on collagen I under TGF-β1 stimulation to 60%. This decrease was even more pronounced on collagen IV (40%). On the contrary, VIM expression was unaffected by the tested treatments. Both collagens in combination with EGF induced EMT-like changes in MDA-MB-468, but EGF was definitely the main cause driving EMT (Supplementary data, Figure S2), as its single treatment exhibited the strongest effect on CDH1 and VIM levels.

Surprisingly, EMT-induction experiments in the HCC1954 cell line revealed that vimentin protein increment was independent of collagen type when co-treated with TGF-β1 (Figure 4e). Thus, the data underline the necessity of collagen coatings for TGF-β1 to drive EMT. Again, it was mostly vimentin expression that changed, whereas E-cadherin levels hardly differed from untreated samples. EGF stimulation did not lead to phenotypic changes regardless of the collagen type the cells were seeded on (Figure 4d).

#### 2.2.2. Morphology

**Figure 5:**
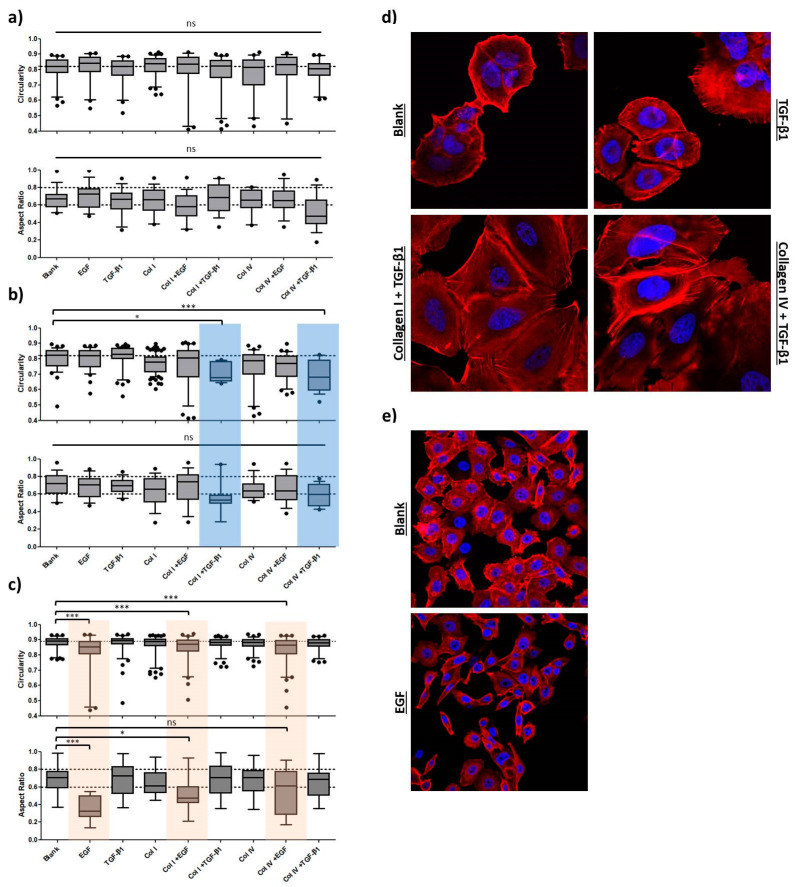
Morphological analysis as part of the EMT-induction study. Confocal image analysis of fixed samples treated for 72 h. Cells were subjected to multiple treatments as indicated. Nuclear circularity C_N_ (upper panel) and cellular aspect ratio A_R_ (lower panel) were calculated using Fiji software. Data are presented as whisker plots with 10–90 percentiles. One-way ANOVA with Dunnett’s Multiple Comparison Test was performed in GraphPad Prism software to calculate *p*-values (ns: *p* > 0.05, * *p* < 0.05, *** *p* < 0.001). (**a**) Shape factor analysis of MCF7 cells treated with growth factors and/or grown on collagen demonstrated no significant changes in cellular morphologies. (**b**) Shape factor analysis of HCC1954 cell line showed significant alterations as part of a combinatorial treatment of TGF-β1 with collagen coatings (highlighted in blue). (**c**) Shape-factor analysis of MDA-MB-468 cells revealed significant decrease of C_N_ and A_R_ for EGF stimulation (highlighted in orange). (**d**) Confocal images of fixed cells were recorded with a 63× objective. Nuclei are shown in blue (DAPI) and the actin cytoskeleton in red (Phalloidin-TRITC). Cell growth of HCC1954 cells subjected to TGF-β1 treatment either grown on conventional glass coverslips or on collagen-coated dishes. Combinatorial treatment resulted in restructuring of the cytoskeleton and stress fiber formation as part of the EMT program. (**e**) Confocal images of fixed cells were recorded with a 40× objective. MDA-MB-468 cell growth comparison between untreated cells (Blank) and EGF-treated cells. EGF stimulation resulted in loosened cell–cell junctions. Cells disseminated from epithelial clusters and exhibited more elongated shapes compared to untreated cells.

The re-organization of the cytoskeleton as part of the EMT program converts cells into a phenotype with increased motility and a spindle-like shape. As shown above, the morphology of mesenchymal MDA-MB-231 cells significantly differed from the other three cell lines based on aspect ratio A_R_ and nuclear circularity C_N_. EMT induction is expected to result in A_R_ and C_N_ approaching values comparable to those of MDA-MB-231 cells. The morphological analysis of confocal images is depicted in Figure 5.

Neither the A_R_ nor the C_N_ were significantly altered in the induction study for the MCF7 cell line (Figure 5a). The A_R_ of the sample co-treated with collagen IV and TGF-β1 (0.520 ± 0.195) showed the strongest deviation from the untreated cells (0.659 ± 0.122), whereas all C_N_ values remained comparable to control cells (0.801 ± 0.079).

Morphological assessment of confocal images of HCC1954 cells (Figure 5b) displayed a significant decrease in nuclear circularity for TGF-β1-stimulated samples seeded on collagen I (*p* < 0.05) and collagen IV (*p* < 0.001). Remarkably, the A_R_ of the latter samples also strongly deviated from the untreated cells (0.801 ± 0.077), at 0.709 ± 0.062 and 0.689 ± 0.099, respectively, although this difference was not statistically significant. As can be seen in Figure 5d, cells treated solely with TGF-β1 had the same shape and cytoskeletal architecture as untreated cells. Once grown on either collagen I or collagen IV, actin bundles (stress fibers) were formed, and the cellular outgrowth and shape differed from the control.

Furthermore, analysis of the MDA-MB-468 cells revealed that C_N_ was significantly reduced for all samples treated with EGF (*p* < 0.001) in comparison to the untreated cells (Figure 5c). Similar to what has been observed in HCC1954, a decrease in C_N_ was accompanied by a significant reduction in the aspect ratio, most prominently for EGF stimulation alone (control: 0.695 ± 0.161 vs. EGF: 0.358 ± 0.129). Cell–cell contacts diminished, and cellular shape became elongated when exposed to EGF (Figure 5e). A_R_ and C_N_ values of the other treatments varied marginally from those of untreated cells. In another experiment (Supplementary data, Figure S3), we showed that the degree of morphological change depended on the concentration of EGF. Applying concentrations from 10 ng/mL to up to 50 ng/mL during a 72 h incubation period resulted in continuous reduction in A_R_ and C_N_ values, implying a more pronounced EMT induction for higher concentrations of the growth factor. However, the highest concentration of EGF (100 ng/mL) reverted the effects on A_R_ and C_N_, and cells exhibited rounded shapes similar to untreated cells. Consequently, there might be an optimal range of EGF concentration to induce EMT-like changes.

#### 2.2.3. Migration

Based on our previous findings, we conducted migration assays with a focus on treatments that have shown considerable effects on EMT-marker expression and morphology during EMT induction.

**Figure 6:**
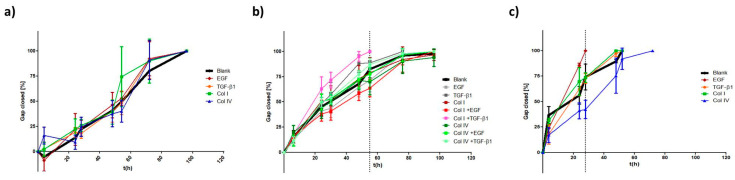
Influence of EMT induction on migratory properties of BC cell lines. Ibidi^®^ migration assay of MCF7 (**a**), HCC1954 (**b**) and MDA-MB-468 (**c**) are shown after 48 h incubation. Cells were subjected to multiple treatments as indicated. Migration is analyzed by comparing the percentage of gap closure [%] over time [h]. Dotted lines depict time point of 100% gap closure of faster moving cells, highlighting treatments that influence cellular migration.

Apart from moderate changes in CDH1 levels after exposure to TGF-β1 or collagen I/IV, MCF7 cells mainly retained their phenotype. According to the migration analysis depicted in Figure 6a, these changes were insufficient to alter the migratory behavior of MCF7.

Additionally, combinatorial treatments for HCC1954 cells (Figure 6b) were included. Interestingly, coaction of TGF-β1 and collagen I enhanced cellular motility. Time for completing gap closure was reduced to 56 h (vs. 96 h for untreated cells). No other treatment provoked similar changes. Even the combination of TGF-β1 with collagen IV had no impact on migration, in contrast to the morphological changes discussed above.

A striking effect on migration was observed for EGF treatment in MDA-MB-468 cells. Its presence accelerated cellular migration almost twofold. The time necessary to close the gap was reduced by 46% (28 h vs. 52 h). Of note, the migration under exposure to collagen IV was strongly decelerated. Cells required 72 h for a 100% gap closure. TGF-β1 and collagen I had no influence on the cellular motility in MDA-MB-468 (Figure 6c), which is in line with the lack of morphological effects discussed above.

### 2.3. EMT-Phenotyping Model and Its Application

**Figure 7:**
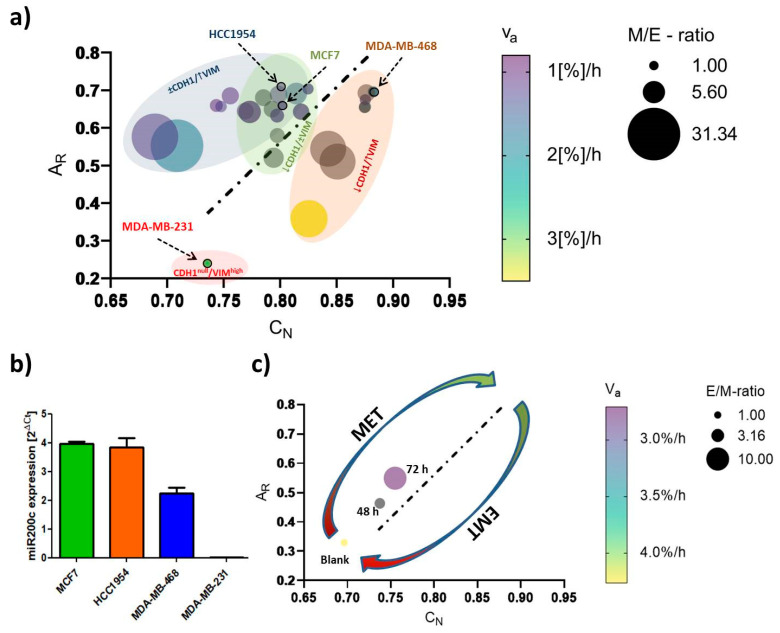
(**a**) EMT-phenotyping model for breast cancer. Summary of the data obtained from the cell lines merging EMT-marker protein levels, morphological and migration analysis; (**b**) miR200c expression levels of the four cell lines; (**c**) applying miR-200c induction in MDA-MB-231 cells to test the EMT model. Data were extracted after 48 h and 72 h of DOX-dependent miR200c induction and compared to untreated (Blank) cells. Mean values of nuclear circularity C_N_, cellular aspect ratio A_R_, apparent velocity *ν_a_* ([%] gap-closure per hour) and E/M- or M/E-ratio (= mesenchymal/epithelial-ratio on the protein level (VIM/CDH1)) were plotted in a multiple variable bubble plot. Results of the EMT induction study are included in (**a**). A M/E-ratio (E/M) of 1 indicates untreated control cells of each cell line, and the respective controls are further indicated by an arrow. With increasing size of the bubbles, cells approach a protein setup phenotypic of mesenchymal (**a**) or epithelial (**c**) cells. Colors of the bubbles indicate the migratory behavior, with yellow representing the fastest moving cells. Grey bubbles were not attributed to apparent velocity. Ellipsoid, colored accentuations depict cell-line-specific phenotypic changes, with green representing MCF7 cells, purple, HCC1954 cells, orange, the MDA-MB-468 cell line and red, the untreated sample of MDA-MB-231 cell line. The black dotted line shows the linear regression diagonal (A_R_/C_N_) of the control cells (y = 2.935 × −1.787; R^2^ = 0.62).

As indicated in Figure 7, a phenotyping model to monitor EMT status and EMT-like changes is proposed. It is essentially based on C_N_ and A_R_. We expanded it by queuing data from the aforementioned cellular EMT-protein marker settings and migratory behavior. Providing the M/E-ratio (normalized mesenchymal vimentin protein levels (M) divided by normalized E-cadherin protein levels (E)) for each treatment enabled us to correlate the magnitude of phenotypic transition on the protein level with the image-based shape analysis. Cellular motility, expressed as the apparent velocity *ν_a_* ([%] gap-closure per hour) further contributed to this model. Accordingly, rounded, slowly migrating epithelial-like cells are found in the upper right corner, and highly motile, mesenchymal-like cells are in the lower left corner of the plot. Alongside the linear regression diagonal (A_R_/C_N_) of the control cells (dotted line), the cellular phenotype is transiting from a low-EMT to a high-EMT status. A decrease in E-cadherin level is accompanied by a lowering of the A_R_, whereas the deformation of the nucleus (C_N_ values) showed a better correlation with vimentin upregulation.

In order to validate the model in a therapeutic context, miR200c (microRNA200c) expression in the four cell lines was quantified. miR200c is known to fulfill a regulatory function regarding the epithelial–mesenchymal state of a cell, as it directly inhibits the activity of pro-EMT transcription factors ZEB1 and ZEB2 [47]. Therefore, miR200c expression is thought to correlate with E-cadherin expression and, consequently, with an epithelial phenotype. Indeed, miRNA expression analysis (Figure 7b) demonstrated high levels of miR200c in epithelial MCF7 and HCC1954 cell lines. Moreover, miR200c expression was about halved in the E/M-hybrid cell line MDA-MB-468 and absent in mesenchymal MDA-MB-231 cells. Hence, we clearly confirmed the correlation between miR200c expression levels and the underlying E/M phenotype.

Recently, it was shown that the re-expression of miR200c in vitro partially reverses the mesenchymal phenotype of MDA-MB-231 cancer cells, i.e., leading to a mesenchymal-to-epithelial transition (MET) [47]. Using a miR200c-inducible MDA-MB-231 cell line, we sought to exemplify a therapeutic intervention of targeting EMT, i.e., by inducing MET (Figure 7c). Strikingly, applying the miR200c-inducible cell line for 48 and 72 h to our model, we monitored morphological features to follow the linear regression diagonal (y = 2.935 × −1.787) calculated above. Setting the C_N_-values in the equation predicted A_R_-values of 0.245, 0.387 and 0.438 for 0 h, 48 h and 72 h time points, respectively, which were in relatively good agreement with the actual mean A_R_-values (0.319, 0.421 and 0.546). Concomitantly, the increased E/M-ratio and decreased apparent velocity were indicative of a transition towards a more epithelial phenotype, i.e., for the success of the modeled therapeutic intervention.

## 3. Discussion

Here, a simplified EMT-relevant breast cancer model comprising four breast cancer cell lines was established. The triad of EMT-marker expression, morphology and migration allowed for a reasonable approximation of the present cellular EMT status. Merging their input into a computational model phenotyping EMT in breast cancer can serve as a new platform to support EMT-related research. Moreover, biomimetic collagen coatings were shown to partially (cell-line dependent) but not necessarily influence cellular EMT phenotype upon growth factor stimulation. Surprisingly, in contrast to results reported elsewhere [36], the distinct collagen types chosen to represent the compartmentalization of the mammary gland (DCIS vs. IBC) did not differentially affect the phenotype and phenotypic transitions. Presumably, including in-vivo-like cues such as the three-dimensionality and stiffness of the ECM would help to draw a final conclusion.

Underlining the current understanding of EMT in cancer research [4,5,7,8,14,27,61,62], (high) vimentin expression and absence of E-cadherin determined a fairly migratory phenotype with an elongated shape as was shown for the MDA-MB-231 cell line. Expression of the epithelial protein marker E-cadherin appeared to have a strong impact on morphological features, as the three E-cadherin^+^ cell lines studied here exhibited rounded shapes with A_R_ and C_N_ values close to a value of 1. Further, its expression correlated with an immotile phenotype as long as vimentin was not co-expressed. Indeed, even low levels of vimentin protein could be correlated with increased cellular motility over vimentin-cells, as was demonstrated for the MDA-MB-468 cell line. In parallel to the classification of the intrinsic subtype nomenclature, epithelial characteristics vanished towards more malignant phenotypes, whereas mesenchymal features concentrated in both TNBC cell lines (Figure 2 and Figure 3).

In a recent study, it was reported that Slug-mediated downregulation of E-cadherin together with upregulation of vimentin impaired cellular morphology and increased cellular motility of the non-malignant breast epithelial cell line MCF10A [63]. Cellular circularity, which is interchangeable with the aspect ratio, was strongly decreased. Subsequent RNA interference with vimentin-targeting siRNA not only restored the circular cellular shape but also decelerated cellular motility. The authors attributed vimentin a crucial role in influencing cellular morphology and motility, also because it may directly inhibit E-cadherin expression. In another study on breast cancer, E-cadherin expression was proposed to be obligatory for a round polygon shape [47]. However, the findings of our EMT-induction study suggest that upregulation of vimentin and concomitant downregulation of E-cadherin protein levels drive important morphological changes and strengthen migratory behavior, whereas merely downregulation of E-cadherin, concluded from the MCF7 cell line, was insufficient to significantly alter either of the latter two processes. As seen for MDA-MB-468, EGF stimulation significantly elongated cellular and nuclear morphology as part of a pronounced EMT induction (↓ E-cadherin, ↑↑ vimentin). The A_R_ and C_N_ values of MDA-MB-231^blank^ (A_R_ = 0.240 ± 0.157; C_N_ = 0.735 ± 0.101) and MDA-MB-468^+EGF^ (A_R_ = 0.358 ± 0.129; C_N_ = 0.825 ± 0.102) essentially converged in a concentration-dependent manner (Figure S2), implying a shift from an E/M-hybrid with mostly epithelial characteristics towards a phenotype endowed with dominant mesenchymal functionalities (Figure 5c and Figure 7a). Interestingly, upon EGF stimulation in MDA-MB-468, cellular migration was superior or at least the same as has been observed for untreated MDA-MB-231. It took 24–28 h to close the gap compared to 30–35 h for the already mesenchymal cell line.

Moreover, we successfully transformed HCC1954 cells into a hybrid E/M phenotype, entailing alterations of both shape factors and an acceleration of cellular motility. However, the combinatorial action of collagen and TGF-β1 was required to drive the transition. Similarly, it was reported that cells at the tumor margin partially acquire mesenchymal effector proteins (e.g., vimentin) whilst maintaining cell–cell contacts, phenotypic of epithelial cells [37,45]. A partial EMT can supply cells with elevated motility, but cells concomitantly benefit from survival cues resulting from cell–cell interactions. Indeed, it is believed that a broad spectrum exists of such intermediate states, with the degree of transition depending on the present context [45]. Here, the wound-healing migration assay revealed similar migratory properties of HCC1954 cells under combinatorial treatment of TGF-β1 with collagen I, as was detected for hybrid MDA-MB-468^blank^ cells (56 vs. 50 h). It appears that the strongly elevated vimentin protein level is the main driver of increased cellular motility, as E-cadherin levels remained unaffected. Contradictory to these findings, combinatorial treatment of TGF-β1 with type IV collagen, also shown to upregulate vimentin expression, did not result in increased motility.

We can only speculate about the mechanistic causality explaining how a combinatorial treatment can provoke EMT-like changes while the single components fail to do so. In accordance with our data, Buckley et al. examined TGF-β1-induced EMT in human alveolar epithelial cells (AEC) and found that following TGF-β1 stimulation, EMT induction was enhanced when cells were grown on a collagen I matrix compared to growth on a glass surface [41]. They based their findings on changes in cellular shape factor and cellular stiffness being superior to the simple growth factor treatment and emphasized the role of integrins (“ECM-receptors”) for EMT induction as was reported elsewhere [64]. In their work on EMT in fibrosis, the authors elegantly ruled out the possibility that growth of AECs on the ECM would lead to increased secretion of TGF-β1 which in turn drives EMT. Instead, they provided strong evidence that the αvβ6 integrin, a receptor that binds fibronectin, activates latent TGF-β1 signaling that causes cells to undergo EMT. However, this cannot be the explanation in our case. Firstly, the αvβ6 integrin is not known for binding to any collagen type, and secondly, their observation was independent of TGF-β1 supply. Nevertheless, the literature offers two other explanations for how integrin–growth factor receptor (GFR) interplay may modulate cellular phenotypes [65]. GFR–ligand interactions can lead to cytosolic inside-out integrin receptor activation or changes in the expression pattern of integrin subunits [66]. Referring to our data, TGF-β1 signaling may have caused enhanced integrin activation/expression that would have resulted in intensified integrin signaling (through collagen–integrin interaction), ultimately leading to EMT-like changes. On the other hand, integrins may co-opt in GFR signaling cascades. Signals emitting from integrin activation participate in downstream processes of GFR stimulation. Consequently, those kinds of interactions may not be the driving force for EMT, but rather scale up its dimension. Hence, we have to consider that the 48 to 72 h time scope of our EMT induction study was insufficiently long to detect TGF-β1-mediated EMT in HCC1954. However, our findings, together with the cited literature, highlight the evident role of both contextuality of signaling and the tumor microenvironment for EMT induction in vitro. Cellular responses are affected by the combinatorial action of growth factors and ECM components, requiring future 2D EMT research to consider this context-dependency by applying similar experimental setups. Such combinatorial actions might induce EMT in DCISs, helping to overcome the physiological barrier, i.e., the basement membrane, whilst sustaining a (more) mesenchymal phenotype during stromal invasion.

Confocal imaging combined with image data analysis has been proven to be a powerful tool for addressing many kinds of biological questions. In recent years, imaging has become increasingly relevant for studies on “phenomics”, the quantification of the plurality of phenotypes that fully characterizes an organism [67]. Collective cellular properties such as cellular and subcellular morphologies are the result of genotypic expression patterns, whose quantification is readily susceptible via image data analysis [68]. In the context of EMT, a thoroughly planed study by Wang et al. stunningly demonstrated how live-cell imaging with subsequent deep image analysis disclosed heterogeneous transition dynamics upon TGF-β stimulation within one cell line [69]. They described a cell state in a 309-dimensional composite feature space of cell morphology and vimentin texture features and further revealed that spatiotemporal vimentin distribution allows for recording phenotypic alteration. The EMT-phenotyping model presented here is rather a snapshot approach, as the comparison of shape factors (C_N_, A_R_) was conducted at a specific time-point. As indicated in Figure 7, the morphological features coincided with EMT-marker expression and cellular motility. It is noteworthy that this computational model not only spans EMT phenotype and phenotypic changes but presumably permits the differentiation between cell-line-specific transition dynamics as indicated by the elliptic, colored areas in Figure 7a. To exemplify how this model can be used, we applied it to a miR200c-inducible MDA-MB-231 cell line (Figure 7c and Figure S4). miR200c expression, which is absent in untreated MDA-MB-231 cells, correlated with an epithelial phenotype (Figure 7b). The height of miR200c expression levels further discriminated between fully epithelial and partial epithelial characteristics, and thus can be included as an additional indicator to define the present E/M-phenotype. Induction of miR200c in the MDA-MB-231 cell line resulted in MET, resembling a potential therapeutic intervention targeting EMT. The success of miRNA induction after 48 h and 72 h resulting in increased nuclear circularity—and cellular aspect ratios closer to 1.0—can be appreciated as the shift from the left to the right alongside the regression diagonal. Simultaneously, the increasing E/M-ratio (= reciprocal M/E-ratio) and the decrease in *ν_a_* are testifying to MET. Hence, the model was able to resolve distinct intermediate EMT phenotypes that occurred during MET. It may therefore be sufficient to analyze one of the aforementioned shape factors in order to predict the cellular E/M character as well as cellular motility. Thus, our model can estimate the impact of therapeutic approaches that target EMT-relevant factors on the EMT phenotype and may serve as an indicator to decide on new drug candidates during screening processes.

## 4. Materials and Methods

***Materials and cell culture***: Formaldehyde solution (≥36%), 4′,6–diamidino–2-phenylindole dihydrochloride (DAPI), FluorSave reagent, DNase I (recombinant, RNase-free), cOmplete™, EDTA-free Protease Inhibitor Cocktail, Phosphatase Inhibitor Cocktail 2, RIPA buffer, Tris-buffered saline powder, Ponceau S Stain, Tween 20, Amersham™ Protran^®^ Western-Blotting-Membrane (nitrocellulose) and for cell culture, Eagle’s Minimum Essential Medium (EMEM), RPMI-1640 Medium, Dulbecco’s modified eagle’s medium (DMEM), fetal bovine serum (FBS), Penicillin-Streptomycin (Pen/Strep) solution, Dulbecco’s phosphate-buffered saline (PBS), trypsin-EDTA solution 0.05 and 0.25%, 200 mM of L-glutamine solution and dimethyl sulfoxide (DMSO) were purchased from Sigma-Aldrich (Taufkirchen, Germany). GAPDH Monoclonal Antibody (ZG003), Pierce™ BCA Protein Assay Kit, Novex™ 10% Tris-Glycine Mini Gels (WedgeWell™ format, 15-well), Novex™ Value™ 4–20% Tris-Glycine Mini Gels (1.0 mm, 10-well), Page Ruler™ Plus Prestained Protein Ladder 10 to 250 kDa, Tris Glycin transfer buffer, SuperSignal™ West Pico PLUS Chemiluminescent Substrate, Rhodamine Phalloidin, High capacity cDNA synthesis kit, Power SYBR™ Green PCR Master Mix, PureLink™ RNA Mini Kit, and for cell culture, Leibovitz’s L-15 Medium and MEM Non-Essential Amino Acids Solution (100×) were purchased from Thermo Fisher Scientific (Waltham, MA, USA). Hs_CDH1_Primer Assay (QT00080143), Hs_VIM_Primer Assay (QT00095795) and Hs_GAPDH_1_SG QuantiTect Primer Assay (QT00079247) were purchased from Qiagen (Hilden, Germany). Collagen I (sc-136154), collagen IV (sc-29010), m-IgGκ BP-HRP (sc-516102), E-cadherin Antibody (G-10) and Vimentin Antibody (V9) were ordered from Santa Cruz Biotechnology (Dallas, TX, USA). Rotiphorese 10× SDS–Page, Rotilabo^®^-Blotting Papers and Methanol (blotting grade) were purchased from Carl Roth (Karlsruhe, Germany). rh-TGF-β 1 (Transforming Growth Factor beta 1) and rh-EGF (Epidermal Growth Factor) were purchased from ImmunoTools (Friesoythe, Germany). HyClone trypan blue solution 0.4% in phosphate-buffered saline was obtained from FisherScientific (Hampton, NH, USA). Culture-Insert 2 Well in µ-Dish 35 mm was purchased from Ibidi (Gräfelfing, Germany). Laemmli loading buffer (4×) was purchased from VWR (Allison Park, PA, USA).

MCF7 (and MCF7 miR200c_KO) cells, a Luminal A breast cancer cell line, were cultured in EMEM supplemented with 10% FBS, 1× Pen/Strep, 1× MEM Non-Essential Amino Acids Solution and 2 mM glutamine. The HER2-positive breast cancer cell line HCC1954 was grown in RPMI-1640 Medium supplemented with 10% FBS and 1× Pen/Strep. MDA-MB-231 (and MDA-MB-231 i-miR200c) cells, a triple-negative breast cancer (TNBC) cell line, were cultured in high glucose (4500 mg/L) DMEM; 10% FBS, 1× Pen/Strep and 2 mM glutamine were added to the medium. The miRNA induction medium is equipped with 5 µg/mL doxycycline hydrochloride as described elsewhere [47]. The latter three cell lines were cultured in a humidified atmosphere with 5% CO_2_ at 37 °C. The second TNBC cell line, MDA-MB-468, was grown in L-15 Medium supplemented with 20% FBS and 1× Pen/Strep. Those cells were held in a humidified incubator with 0% CO_2_ at 37 °C.

***EMT marker—gene expression analysis***: To compare EMT-marker RNA expression among the four cell lines, RT-qPCR was performed. From each cell line, 200,000 cells were seeded in a 6-well plate and cultured for 24 h. After incubation, cells were harvested and total RNA was isolated using the PureLink RNA mini kit according to the manufacturer’s protocol with additional DNAse I digestion. Subsequently, 1000 ng of RNA was used to synthesize cDNA using the high-capacity cDNA synthesis kit. In the following, E-cadherin- and vimentin-specific primers were used to amplify and quantify RNA using Power SYBR™ Green PCR Master Mix and the qTOWER real-time PCR thermal cycler (Analytik Jena, Jena, Germany). C_t_ values were normalized to GAPDH RNA expression and ΔC_t_ values were calculated for the comparison.

In order to quantify the miR200c expression levels, RNA was isolated using the peqGOLD Micro RNA kit (Peqlab Biotechnology GmbH, Erlangen, Germany), according to the manufacturer’s protocol. cDNA was synthesized with the qScript microRNA cDNA synthesis kit (Quantabio, Beverly, MA, USA). Since microRNAs are not polyadenylated, the polyA tailing reaction was performed by mixing 1 µg of RNA, 2 µL of Poly(A) Tailing Buffer, 1 µL Poly(A) polymerase and nuclease-free water up to 10 µL and incubating for 60 min at 37 °C followed by 5 min at 70 °C. Subsequently, 9 µL of microRNA cDNA reaction mix was added to 1 µL reverse transcriptase and incubated for 20 min at 42 °C, plus 5 min at 85 °C. RT-qPCR was performed in triplicate. The microRNA-191 was used as a housekeeper and each sample was analyzed in triplicate.

***EMT marker—protein level analysis***: To define the EMT status, protein levels of CDH1 and VIM of the 4 cell lines were analyzed via Western blotting. From each cell line, 300,000 cells were seeded in a 6-well plate and cultured for 24 h. Total protein extract was isolated after incubation. Briefly, cells were washed 3 times with PBS prior to cell lyses. To each well, 70 µL of proteinase- and phosphatase-inhibitor containing RIPA buffer was added, and cells were kept on ice for 30 min. Then, wells were thoroughly scraped, and the extracts were transferred into 1.5 mL Eppendorf tubes. After a 10 min centrifugation step at 4 °C, total protein concentration was assessed according to the manufacturer’s protocol (Pierce™ (Appleton, WI, USA) BCA Protein Assay Kit). Gels were loaded with 30 µg protein per sample, and electrophoresis was run for 90 min at 120 mV. Subsequent to 1 h of protein transfer at 100 mV, blots were washed, blocked and incubated overnight using E-cadherin-, vimentin- and GAPDH-specific antibodies. HRP-bound secondary antibody was added for 1 h under exclusion of light before blots were developed.

As part of the EMT induction study, we first optimized the time points to extract protein data. It should be noted that cell lines do not facultatively perform EMT and may exhibit different “transition dynamics” (fast vs. slow) upon GF treatment. Therefore, we chose an optimized time point (72 h) and GF concentrations that allowed us to detect EMT-like changes at the protein level in the cell lines used.

To do so, 300,000 cells were seeded to attach for 4 h. Samples included untreated cells, cells stimulated with growth factors and cells grown in collagen-coated wells (+/− growth factors). Afterwards, control cells and cells growing only on collagen were washed with PBS, and pre-warmed medium was refilled. At this step, growth factors were included. Samples were either supplied with 10 ng/mL of TGF-β1 or 25 ng/mL EGF or both. Collagen I and IV coatings (2 µg collagen/cm^2^) of the respective wells were prepared following the manufacturer’s protocol in advance of the seeding. After 72 h incubation, samples were subjected to the aforementioned Western blotting protocol, and E-cadherin and vimentin protein levels were normalized to GAPDH-housekeeping protein level using Image Lab™ software Version 6.0.1 (Bio-Rad Laboratories, Hercules, CA, USA).

***Confocal scanning microscopy—morphological analysis***: Confocal image analysis was used to assess and quantify morphological differences between cell lines and treatments. The experimental setup was performed as described above. Briefly, sterile glass coverslips were distributed onto a 24-well plate. Collagen coatings were applied to the respective wells. Thereafter, 40,000 cells were seeded and attached for 4 h. After the growth factor treatment, cells were incubated for 72 h. Then, wells were washed 3 times with PBS before cells were fixed for 15 min with a 4% formaldehyde solution. To stain the actin cytoskeleton, cells were incubated with 8.25 µM rhodamine phalloidin solution for 40 min and then washed another 3 times with PBS. Nuclei staining was achieved by 10 min incubation with a 0.5 µg/mL DAPI solution. Finally, after an additional washing step, samples were mounted on glass slides using FluorSave and stored at 4 °C until the next day. Fluorescence images were acquired using a laser scanning microscope (Leica SP8 inverted, Software: LAS X, Leica microsystems GmbH, Wetzlar, Germany) equipped with an HC PL APO CS2 40×/1.30 and 63×/1.40 oil immersion objective. Diode lasers (405 nm) and a semiconductor laser OPSL (552 nm) were chosen for excitation, and emission was detected in blue (PMT1: 410–520 nm) and yellow (PMT2: 560–760 nm), respectively. Images were further processed with Fiji image analysis software [70]. Nuclear circularity was calculated as $C_{N}=\frac{4\pi A}{P^{2}}$, and the cellular aspect ratio as $A_{R}=\frac{d_{min}}{d_{max}}$. The axes (d_min_ and d_max_) were drawn manually.

**Figure 8:**
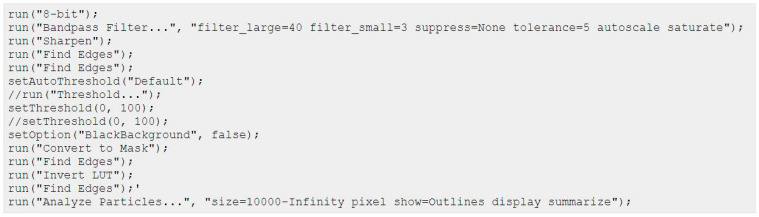
Custom-made macro script used in Fiji software to analyze cell free area.

***Ibidi^®^ migration assay***: Migratory properties were analyzed as follows. To define the cellular EMT status, 25,000 cells of each cell line were seeded in both wells of the Ibidi culture insert. After 24 h, the inserts were carefully removed and the time until gap closure in between the two wells was monitored for up to 120 h, using a Keyence BZ81000 Fluorescence microscope (Keyence, Osaka, Japan). Three to four pictures of different parts of the gaps were taken at each time point. The cell-free area was analyzed based on a custom-made macro within Fiji imaging software (Figure 8):

The percentage gap closure was calculated according to the following equation:

$$\mathrm{Gap}\;\mathrm{closure}\;\left[\%\right]=\left(1-\frac{\mathrm{cell}\;\mathrm{free}\;\mathrm{area}\;\mathrm{at}\;t_{h}}{\mathrm{cell}\;\mathrm{free}\;\mathrm{area}\;\mathrm{at}\;t_{0}}\right)\times 100$$

As part of the EMT induction study, cells were seeded at a density of 15,000 cells per well. Collagen coatings were prepared in advance. Similar to what has been described for the protein analysis, growth factors were supplemented after cell-attachment. Samples were incubated for 48 h before the culture inserts were removed and migration was analyzed.

## 5. Conclusions

The epithelial-to-mesenchymal transition is an ambivalent issue in the field of cancer research, particularly when it comes to its impact on breast cancer metastasis and the transition from DCIS to IBC. Studies that show the independence of metastasis from EMT-phenotypic alterations are scrutinizing the importance of EMT [39,71,72]. Nevertheless, the concept of pEMT has broadened its interpretation, and the increasing amount of literature assessing EMT from other perspectives (e.g., epigenetics, phenomics, biomechanics) is revitalizing the field of EMT. Even conventional cell culture setups enable the study of more complex interrelations such as the contextual EMT induction presented here, which was partially dependent on the combinatorial action of collagen and soluble growth factors. Establishing a more biosimilar context and illuminating EMT from different angles provided us with data allowing for our own interpretation of phenotypic changes during EMT. The devised model contributes to a better understanding of EMT in breast cancer and potentially bears relevance for therapeutic applications.

## Data Availability

Data are available from the authors upon request.

## Supplementary Materials

The supporting information can be downloaded at: https://www.mdpi.com/article/10.3390/ijms24097757/s1.
