# Supplementary material for: An In Vitro Approach to Model EMT in Breast Cancer
Source: Int J Mol Sci. 2023 Apr 24;24(9):7757. doi: 10.3390/ijms24097757 (PMC10177865; doi:10.3390/ijms24097757)
Supplement: Supplementary file 1 [file ijms-24-07757-s001.zip › ijms-2299446-supplementary.pdf]

International Journal of *Molecular Sciences*

MDPI

Supplement

An In-Vitro Approach to Model EMT in Breast Cancer

Lorenz Isert [1], Aditi Mehta [1], Gabriele Loiudice [1], Altea Oliva [2], Andreas Roidl [2] and Olivia Merkel [1,*]

[1] Pharmaceutical Technology and Biopharmacy, LMU Munich, Butenandtstr. 5-13, 81377 Munich, Germany
[2] Pharmaceutical Biotechnology, LMU Munich, Butenandtstr. 5-13, 81377 Munich, Germany
* Correspondence: olivia.merkel@lmu.de

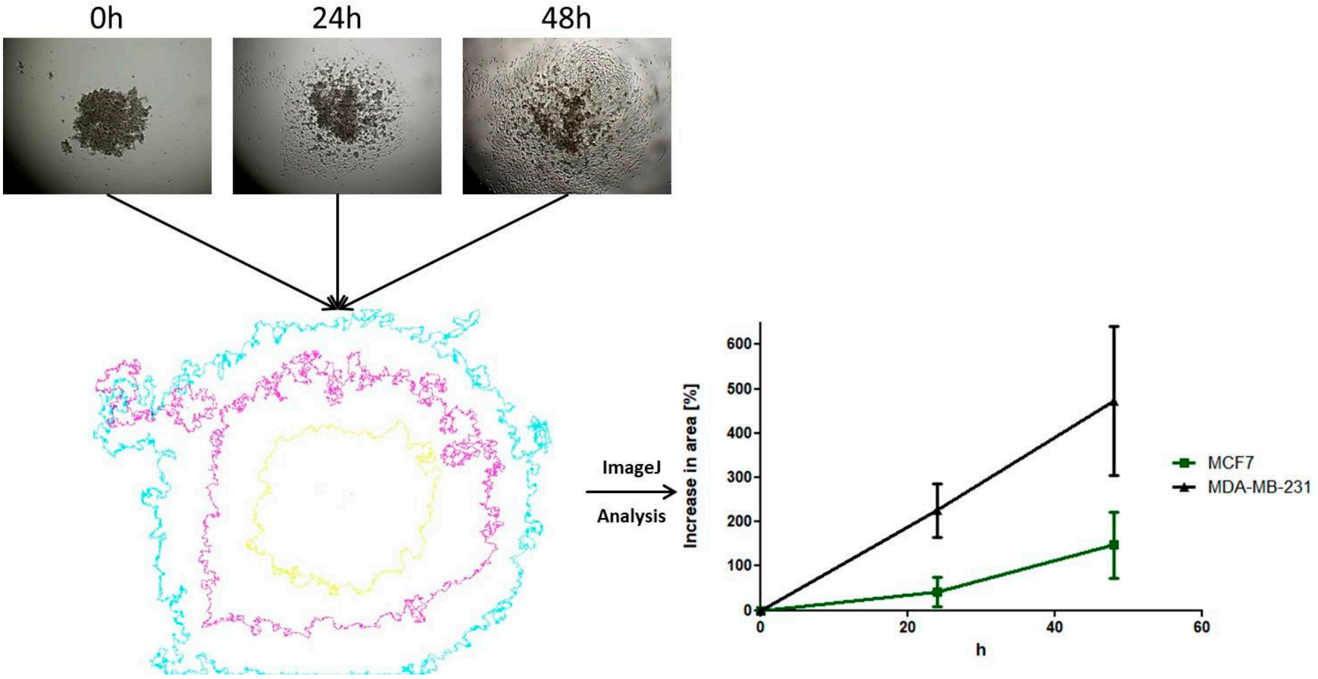

Supplementary Figure S1. Spheroid-based migration assay of MCF7 and MDA-MB-231 cell lines. Spheroids from a hanging-drop cell culture were transferred into a 96-well plate and its spreading was monitored after 24 h and 48 h by imaging and further analysed by Fiji image analysis software. Spreading was calculated as increase in cell area [%] in comparison to cell area at time point t = 0.

Citation: Isert, L.; Mehta, A.; Loiudice, G.; Oliva, A.; Roidl, A.; Merkel, O. An In Vitro Approach to Model EMT in Breast Cancer. *Int. J. Mol. Sci.* **2023**, *24*, 7757. https://doi.org/10.3390/ijms24097757

Academic Editor: Daniela Grimm

Received: 7 March 2023
Revised: 12 April 2023
Accepted: 18 April 2023
Published: 24 April 2023

Copyright: © 2023 by the authors. Submitted for possible open access publication under the terms and conditions of the Creative Commons Attribution (CC BY) license (https://creativecommons.org/licenses/by/4.0/).

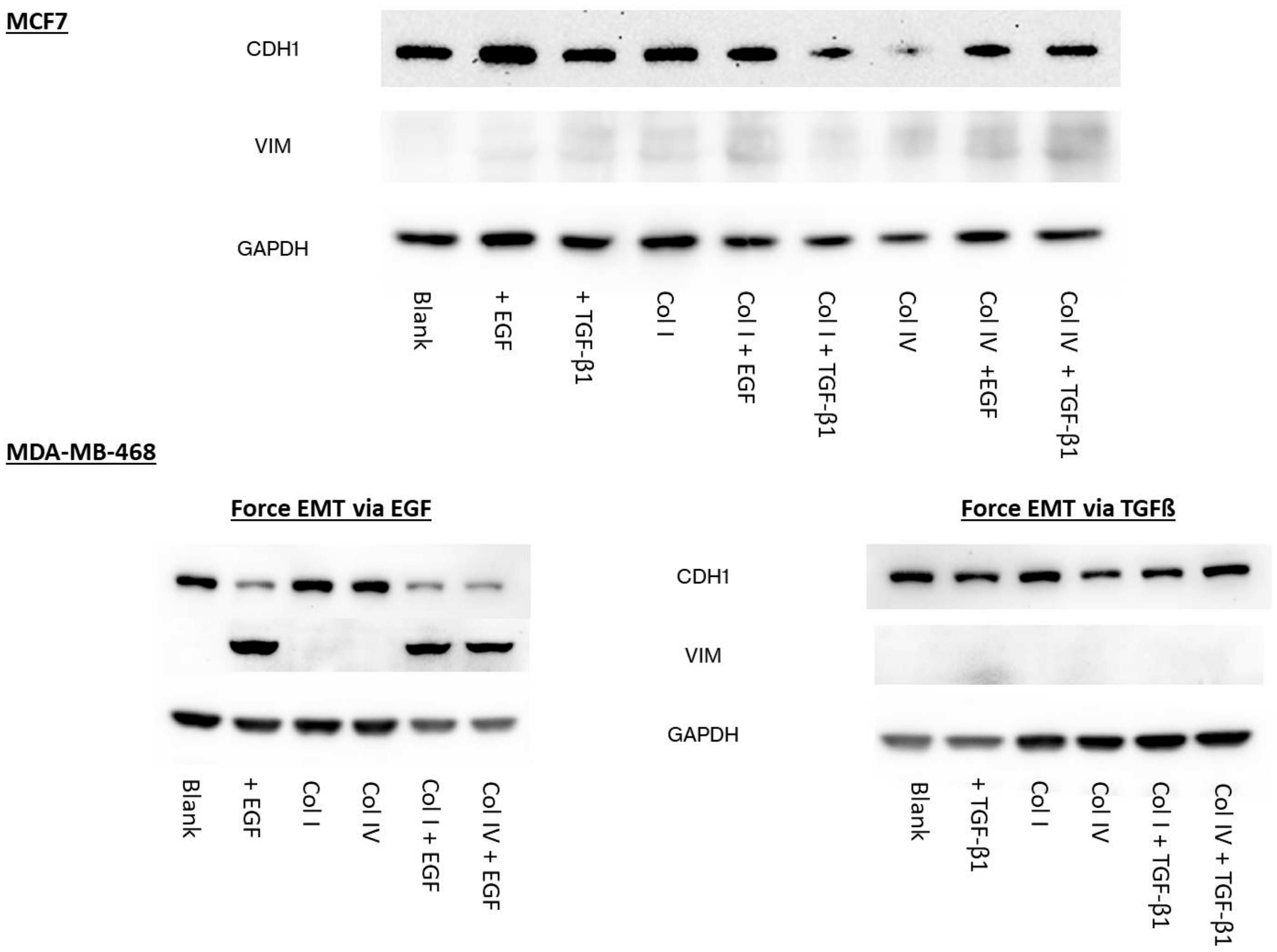

Supplementary Figure S2. Combinatorial treatment of growth factors and collagens. Protein levels of E-Cadherin, vimentin and GAPDH (housekeeping) were assessed via Western blotting from 30 μg of the total protein extracts of MCF7 and MDA-MB-468 cell line. Cells were subjected to multiple treatments as indicated. MCF7 cells dramatically decreased in E-cadherin protein levels upon growth on collagen IV and when grown on collagen I treated with TGF-β1. EGF stimulation induced EMT-like changes in MDA-MB-468.

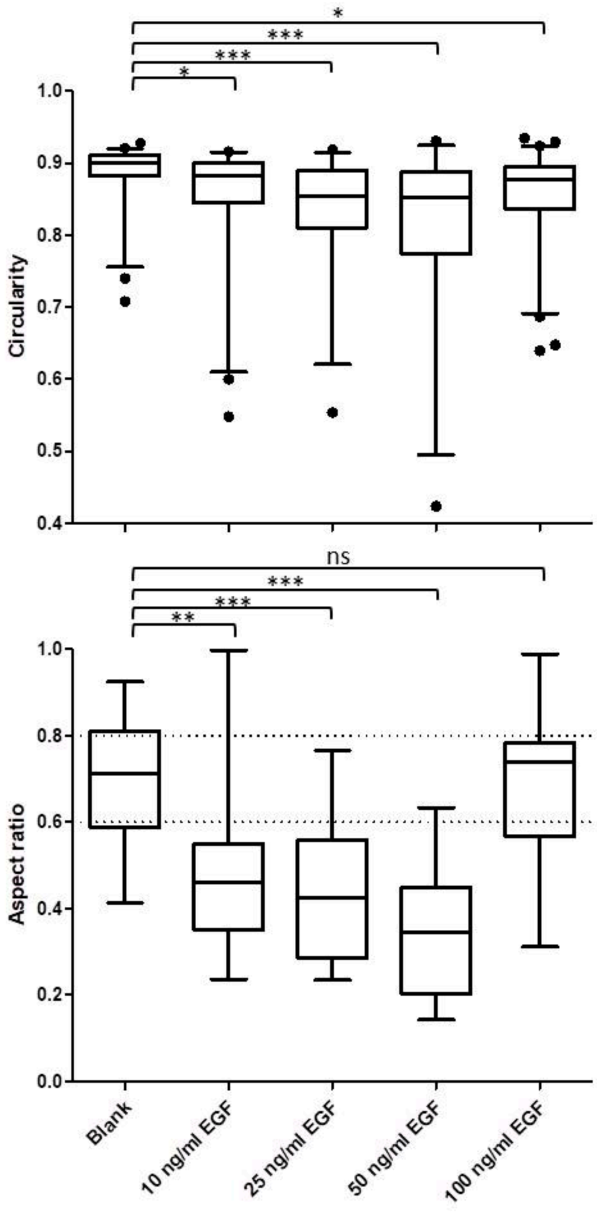

Supplementary Figure S3. EGF-concentration-dependant changes of morphological features in MDA-MB-468. Confocal image analysis of fixed samples treated for 72 h. Cells were treated with increasing concentration of EGF reaching from 0 ng/ml (Blank) to 100 ng/ml. Nuclear circularity C_N (upper panel) and cellular aspect ratio A_R (lower panel) were calculated using the Fiji software. Data is presented as Whiskers plot with 10-90 percentiles. One-way ANOVA with Dunnett's Multiple Comparison Test was performed in GraphPad Prism software to calculate p-values at 95% confidence interval. Morphological features gradually decreased in a concentration-dependent manner.

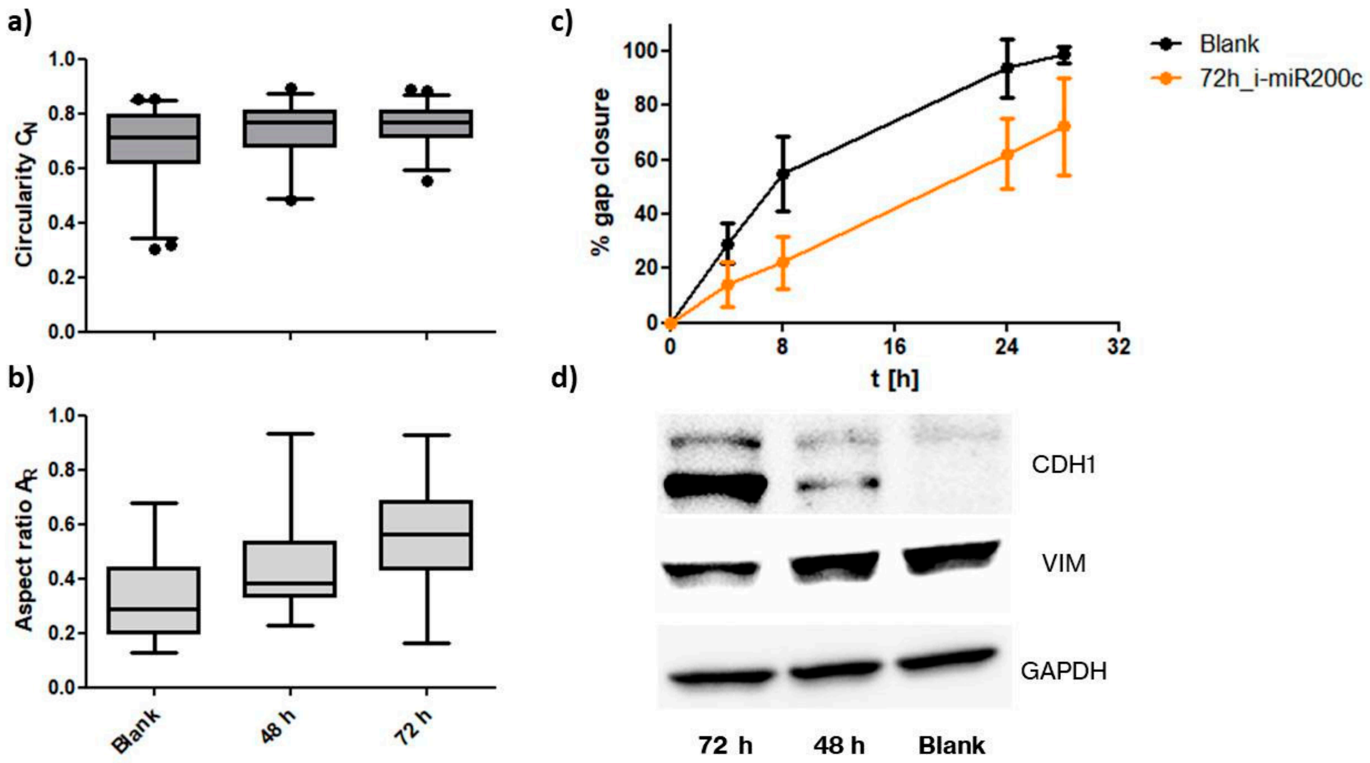

Supplementary Figure S4. miR200c induction in a modified MDA-MB-231 cell line after 48/72 h. Morphological analysis from 63x confocal images of nuclei (**a**) (n > 80) and cytoskeleton (**b**) (n > 20); (**c**) Ibidi® migration assay of untreated/blank cells (black line) and cells induced for 72 h (orange line); (**d**) Protein levels of E-Cadherin (CDH1) and Vimentin (VIM) depending on miRNA induction time
